# The AMP-Activated Protein Kinase Homolog Snf1 Concerts Carbon Utilization, Conidia Production and the Biosynthesis of Secondary Metabolites in the Taxol-Producer *Pestalotiopsis microspora*

**DOI:** 10.3390/genes9020059

**Published:** 2018-01-24

**Authors:** Dan Wang, Yingying Li, Haichuan Wang, Dongsheng Wei, Oren Akhberdi, Yanjie Liu, Biyun Xiang, Xiaoran Hao, Xudong Zhu

**Affiliations:** 1 National Key Program of Microbiology and Department of Microbiology, College of Life Sciences, Nankai University (DMNU), Tianjin 300071, China; nkwangdan@163.com (Da.W.); liyinyingwork@163.com (Yi.L.); wanghaichuansn@163.com (H.W.); weidongsheng@nankai.edu.cn (Do.W.); oren79@163.com (O.A.); 2 Beijing Key Laboratory of Genetic Engineering Drug and Biotechnology, Institute of Biochemistry and Molecular Biology, College of Life Sciences, Beijing Normal University, Beijing 100875, China; yunher@mail.bnu.edu.cn (Ya.L.); xby6024@126.com (B.X.); hxrr_563@163.com (X.H.)

**Keywords:** Snf1/AMPK, secondary metabolism, cell wall integrity, conidiation, *Pestalotiopsis microspora*

## Abstract

Highly conserved, the Snf1/AMPK is a central regulator of carbon metabolism and energy production in the eukaryotes. However, its function in filamentous fungi has not been well established. In this study, we reported functional characterization of Snf1/AMPK in the growth, development and secondary metabolism in the filamentous fungus *Pestalotiopsis microspora*. By deletion of the yeast *SNF1* homolog, we found that it regulated the utilization of carbon sources, e.g., sucrose, demonstrating a conserved function of this kinase in filamentous fungus. Importantly, several novel functions of *SNF1* were unraveled. For instance, the deletion strain displayed remarkable retardation in vegetative growth and pigmentation and produced a diminished number of conidia, even in the presence of the primary carbon source glucose. Deletion of the gene caused damages in the cell wall as shown by its hypersensitivities to Calcofluor white and Congo red, suggesting a critical role of Snf1 in maintaining cell wall integrity. Furthermore, the mutant strain Δ*snf1* was hypersensitive to stress, e.g., osmotic pressure (1 M sorbitol), drug G418 and heat shock, though the mechanism remains to be illustrated. Significantly, disruption of the gene altered the production of secondary metabolites. By high-performance liquid chromatography (HPLC) profiling, we found that Δ*snf1* barely produced secondary metabolites, e.g., the known product pestalotiollide B. This study suggests that Snf1 is a key regulator in filamentous fungus *Pestalotiopsis microspora* concerting carbon metabolism and the filamentous growth, conidiation, cell wall integrity, stress tolerance and the biosynthesis of secondary metabolites.

## 1. Introduction

Adenosine monophosphate (AMP)-activated protein kinase (AMPK) is widely found in eukaryotic cells, which plays diverse, yet critical regulatory roles in the metabolism of nutrients such as sugar, fat and protein and thus concerts the response of the cell to environmental stress [1,2]. Many fungi have the homologs of AMPK, which is one of the subunits in a heterotrimeric protein consisting of one catalytic subunit—α subunit, known as *SNF1* in yeast—and two regulatory subunits—β and γ subunits. Generally, upstream kinases, e.g., Sak1, Tos3 or Elm1, can activate SNF1 protein kinase through phosphorylation of the kinase domains at Thr210 [3,4,5,6,7]. The protein phosphatase complex (Reg1/Glc7), which responses to nutrient signals (such as high concentration of glucose) dephosphorylates SNF1 to inactivated its function [8]. In the baker’s yeast, SNF1 controls the transcription of a large set of genes that covers the cellular processes of carbon sources, gluconeogenesis, respiration, material transport and meiosis. Known as the gauge of energy, SNF1 is generally responsive to the in vivo ratio of adenosine triphosphate (ATP) to AMP to regulate carbon metabolism. *SNF1* mutated strain cannot accumulate glycogen and the intracellular glucose-6-phosphate concentration decreases significantly [9,10,11]. On the other hand, SNF1 complex is one of the key regulators in the responsive processes to stress, e.g., SNF1 activates the heat shock transcription factor (HSF), stress-response transcription factor Msn2 and GATA transcription factor Gln3 [12,13,14,15,16,17]. The general function of the AMPK kinase family is largely conserved over the eukaryotic domains [16,17]. However, its role in filamentous fungi remains largely uncharacterized. 

As a part of our efforts to the understanding of the regulation of secondary metabolism in *Pestalotiopsis microspora*, we identified a homolog of *snf1* in the genome of *P. microspora* NK17. This strain was originally isolated as a producer of taxol-like molecules [18,19]. And an analog of a new class of cholesterol ester transfer protein inhibitor, pestalotiollide B (PB) was also produced by the fungus [20]. Through targeting disruption, we found that *P. microspora snf1* gene was involved in carbon source metabolism and controlled the mycelial growth and development.

It played crucial roles in cell wall biosynthesis, drug resistance and osmotic stress tolerance. Some of the functions are divergent from the baker’s yeast. Our study opens an avenue to explore the function of AMPK in concerting the primary and the secondary metabolism in filamentous fungi.

## 2. Materials and Methods

### 2.1. Strains and Culture Conditions

*P. microspora* NK17, a taxol-producing strain was previously isolated by our laboratory [18]. Its uracil auxotrophic strain, Δura3-NK17, was used as the host for gene manipulation in this study. Unless otherwise specified, fungal strains were grown or maintained in PDA (20% potato, 1.0% dextrose and 1.5% agar) at 25–28 °C, or PDB (PDA without agar) with shaking at 180 rpm, 25–28 °C (MAXQ 6000, Thermo Scientific, Waltham, MA, USA).

### 2.2. Construction of snf1 Mutant and Complemented Strains

The technique, *Agrobacterium tumefaciens*-mediated genetic transformation of NK17, was previously described by Hao et al. [19]. To achieve the deletion of *snf1* (Δ*snf1*), briefly, the deletion vector pOSCAR-URA-Snf1 was constructed through an OSCAR one-step protocol [21]. The selective marker was the gene *pm-ura3* that restores the prototrophic phenotype of uracil biosynthesis. The homologous fragments for targeting of *snf1* were obtained by polymerase chain reaction (PCR) amplification and gel-purified using the AxyPrep DNA Gel Extraction Kit (Axygen, Corning, NY, USA). Thereafter, pOSCAR-URA-Snf1 was delivered into *A. tumefaciens* LBA4404 by electroporation [22]. Then, *A. tumefaciens* carrying pOSCAR-URA-Snf1 was co-cultured with 10^7^ conidia from Δ*ura3-NK17* at 28 °C on IM plate (50 mgL^−1^ uracil and 40 mgL^−1^ acetosyringone) on a nitrocellulose filter. After 2-day incubation, the filter was transferred onto YNB plates supplied with 100 mgL^−1^ cefotaxime. Finally, individual fungal transformants were obtained through single-spore purification. All primers used in this study are listed in Table 1.

For complementation, plasmid pOSCAR-Snf1C was constructed using the BP Clonase reaction and *Hph,* confering hygromycin B resistance, was used as a selective marker. The complementation strain was created by introducing a 4.4-kb fragment containing the wild-type copy of *snf1* into Δ*snf1*. Genomic DNA was used as a template in the amplification of *snf1* by the high-fidelity PCR amplification. Total DNA from each strain was extracted from mycelium grown in 200mL PLB (0.1% lactose to replace of dextrose in PDB) for 4 days, as described by Hao et al. [19]. PCR amplification and Southern blotting were performed to characterize the disruption of *snf1* in Δ*snf1* strain and in the complementation strain *snf1-C* [23].

### 2.3. Analysis of snf1 Roles on Carbon Source Utilization

To determine the effects of alternative carbon source utilization on mycelium growth, equal numbers of conidia (~5 × 10^4^) from NK17, Δ*snf1* and the complementation strain *snf1*-C were placed separately on a YNB (0.17% YNB, 0.5% (NH_4_)_2_SO_4_) supplemented with 2% glucose, 2% sucrose, 2% glycerol, 2% xylose, 2% fructose, 2% maltose and 2% galactose, individually. The plates were then incubated at 28 °C. Three plates were set for each type of sugar.

### 2.4. Quantification of Conidia Production and Mycelia

Conidia were collected from cultures inoculated on PDA at 28 °C for 7 days. The plates were washed twice with sterile distilled water and the concentration of conidia suspension in sterile distilled water was determined by hemocytometer. Total mycelium was isolated from cultures inoculated in PDB, at 28 °C, for 2, 3, 4, 5, 6, 8 and 10 days, respectively. Fresh mycelium was isolated by vacuum filtration and dry mycelium was obtained by vacuum freeze-drying. The weight of the dry mycelium was determined. Triplicates were set in each treatment.

### 2.5. Heat Tolerance Assay

To test the effect of *snf1* on heat tolerance, fresh conidia and mycelium were harvested from cultures inoculated on PDA at 28 °C for six days, or in PDB at 28 °C for three days (*P. microspora* can undergo sporulation readily in liquid culture). Conidia and mycelium were washed with sterile ddH_2_O (double distilled water). Fresh conidia and mycelium were then incubated at 45 °C in water bath for 2 h. Equal numbers of conidia (~5 × 10^4^) or equal volume of mycelium was inoculated on PDA at 28 °C for 168 h. Conidia without heat-shocked served as control. Assays were conducted in triplicates.

### 2.6. Phenotype Observation and Secondary Metabolite Profiling for Δsnf1

Equal numbers of conidia (~5 × 10^4^) from NK17, Δ*snf1* and the complement strain *snf1*-C were inoculated into PDB, or onto PDA plates, respectively, for monitoring phenotypic variation. Each assay was set in triplicate.

To analyze the secondary metabolites, equal numbers of conidia (~5 × 10^5^) from different strains were cultured in 200 mL PDB (in 500-mL flask), 28 °C, with shaking at 180 rpm for 168 h. Mycelium and the liquid phase were separated by centrifugation (Sigma 2-16 KL, Sigma Laborzentrifugen GmbH, Germany) followed by vacuum filtration onto 3M filter paper. The culture supernatant was extracted with an equal volume of ethyl acetate. The organic phase was transferred for evaporation to yield a concentrated residue, which was suspended in 1 mL of methanol. Samples in 20 μL of methanol were injected for high performance liquid chromatography (HPLC) analysis. The device used was the CoM HPLC system (CoMetro Technology, South Plainfeld, NJ, USA). It was conducted at 30 °C, using a C-18 ODS column (4.6 × 250 mm) (Plainfeld) and eluted with methanol/H_2_O. The flow rate was 1 mL/min. The solvent gradient for HPLC was: Solvent A (95% methanol in H_2_O, *v*/*v*) and solvent B (70% methanol in H_2_O, *v*/*v*). The chromatographic data were collected and analyzed digitally using the Agilent EZChrom Elite software (Agilent Technologies, Santa Clara, CA, USA). Yield was determined three times in parallel and variations were calculated as a standard error. Cultured in flasks could reflect vegetative growth and conidiation in liquid culture.

### 2.7. Drug and Stress Sensitivity Assays

For drug and stress sensitivity assays, 5 × 10^4^ conidia from NK17, Δ*snf1* and *snf1*-C were incubated on PDA plates supplemented separately with 0.02% Congo red, 0.8 M KCl, 1 M NaCl, 1 M sorbitol, 2 mM H_2_O_2_, 50 μg/mL G418, 300 ng/mL caspofungin and 50 ng/mL calcofluor white. All cultures were incubated at 28 °C for seven days, in triplicate. 

## 3. Results

### 3.1. Characterization of snf1 Homolog in Pestalotiopsis microspora

**Figure 1:**
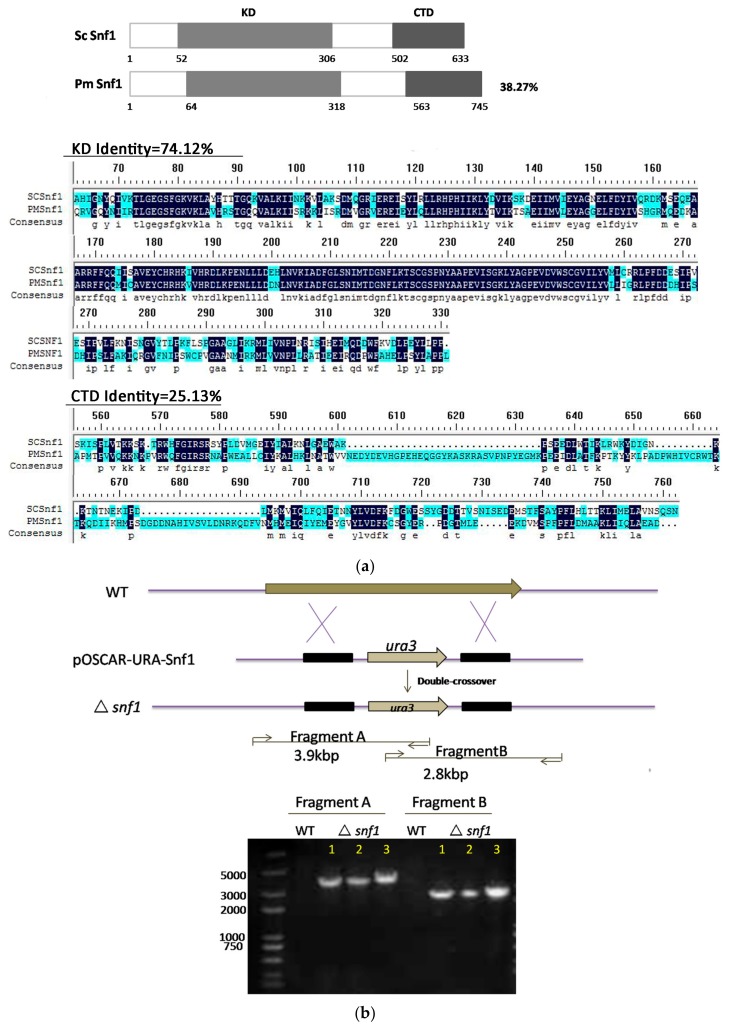

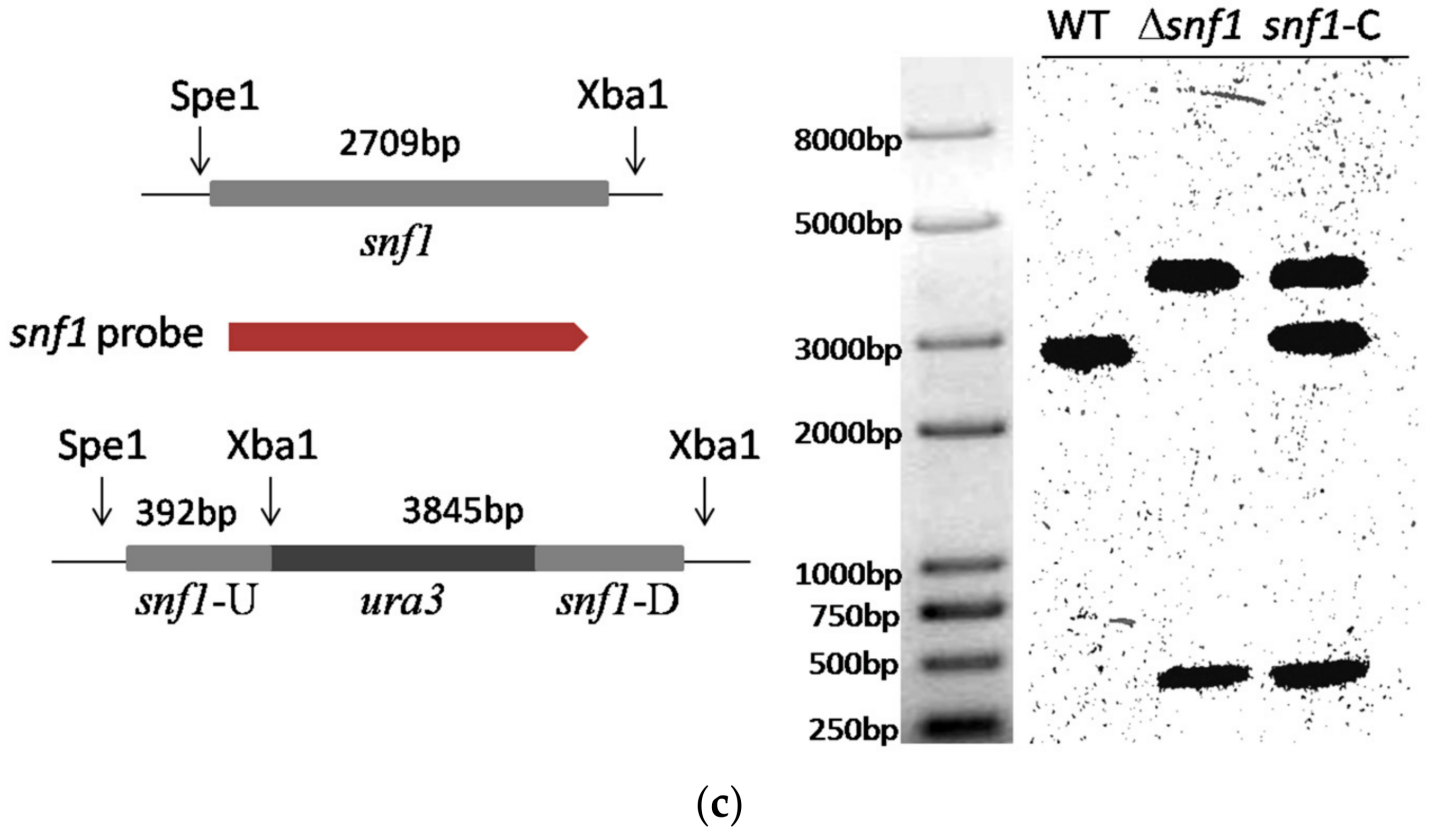
Identification and deletion of *snf1* in *Pestalotiopsis microspora* NK17. (**a**) Schematic diagram of the structure of Snf1 protein from *P. microspora* NK17 and amino-acid sequence (partial sequence) alignments of Snf1 homologs from *Saccharomyces cerevisiae*. Numbers indicate the length and the location of the conserved domain of the protein, including kinase domain (KD) and C-terminal domain (CTD). The overall percentage of amino acid sequence identity with ScSnf1 is shown on the right. The identical amino acids are starred and highlighted in blue and the less conserved amino acids are shown in turquoise; (**b**) The disruption cassette on the vector pOSCAR-URA-Snf1 carried the homologous fragments of *snf1*. Bottom panel showed the polymerase chain reaction (PCR) verification of the deletion in the mutants. Two fragments, 3.9 kb and 2.8 kb, were obtained by PCR amplification for Δ*snf1*, while no band was seen for NK17; (**c**) The left panel, the wild-type copy that was reintroduced in the deleted strain Δ*snf1* was shown. And the probe used in Southern blotting (the right panel) was highlighted in red. Southern blot was shown on the right to confirm the deletion of *snf1*. Genomic DNAs from NK17, Δ*snf1* and the complementation strain *snf1-C* were digested with *Spe* I and *Xba* I. The probe used for Southern blotting was amplified by primers Snf1-up (F) and Snf1-down (R). Two bands on the membrane, at 0.4 and 3.8 kb, were obtained for Δ*snf1*, while in wild-type (WT) NK17, one band of 2.7 kb was observed. In the complementation strain *snf1-C*, there were three bands (0.4, 2.7 and 3.8 kb).

The genome of *P. microspora* NK17 has been sequenced (unpublished data). When searching using the amino acid sequence of Snf1 from *Saccharomyces cerevisiae* as the query, only one homologous protein was found in *P. microspora* NK17 (38.27% identity and designated as *snf1*, GenBank accession no. MG462730). An open reading frame (ORF) of *snf1* in *P. microspora* NK17 was predicted to be 2544 bp in length, encoding a peptide of 745 amino acids (Figure 1a). Structural analysis of Snf1 suggested that this protein contains a catalytic kinase domain (KD) (residues 64-318) and a C-terminal domain (CTD) (563-745 residues). Amino acid sequence alignment of KD (74.12% identity and CTD (25.13% identity) indicated that Snf1 shared high identity with Snf1 homologs in *S. cerevisiae* (Figure 1a). The high similarity among these sequences suggests a conserved function of Snf1 in *P*. *microspora*. 

To investigate the role of *snf1* in *P. microspora* NK17, we made a targeted disruption mutant for *snf1* via homologous recombination. The disruption cassette was described in detail in the Materials and Methods section. The open reading frame (ORF) of Snf1 was replaced by the selection marker (*ura3* gene) (Figure 1b). Three transformants were confirmed as anticipated by PCR amplification and Southern blotting (Figure 1b,c). The transformant No.3 was used for further study. The complementation strain was obtained by way of a procedure described in the Materials and Methods section and verified by Southern blotting (Figure 1c).

### 3.2. Snf1 Is Necessary for Conidiation, Colonial Pigmentation and Vegetative Growth 

**Figure 2:**
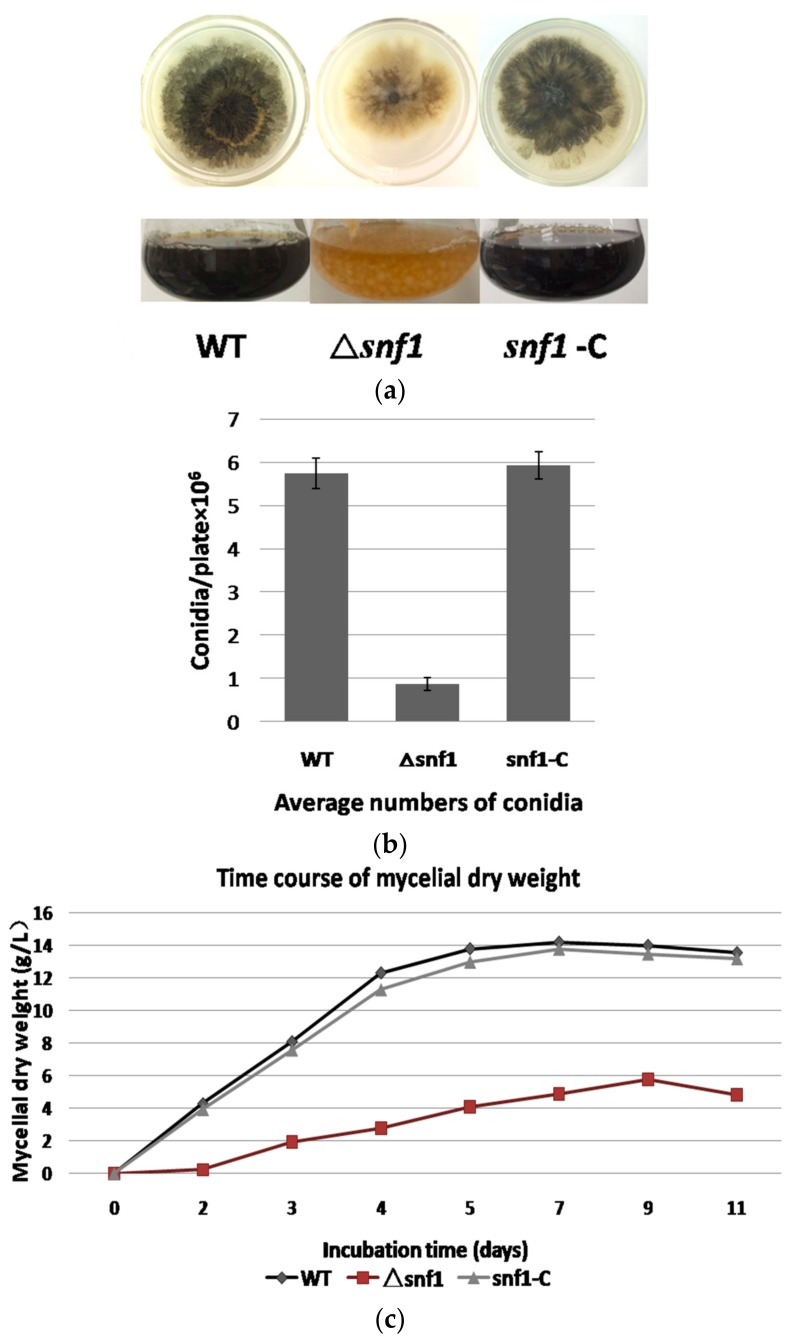
Phenotype of Δ*snf1*. (**a**) Difference in pigmentation and growth rate between the wild type (WT) or the complement strain *snf1-C* and Δ*snf1*. Strains were grown either on PDA or in PDB; (**b**) The number of conidia produced by NK17, Δ*snf1* and the complement strain *snf1*-C was determined as 5.74 ± 0.35 × 10^6^ (*p* < 0.01), 0.87 ± 0.15 × 10^6^ (*p* < 0.01) and 5.74 ± 0.35 × 10^6^ (*p* < 0.01), per plate, respectively. Each strain was incubated at 28 °C for 168 h. Error bars represent standard deviation; (**c**) A growth curve based on the quantity of dry-weight of mycelia for NK17, Δ*snf1* and the complementation strain *snf1*-C in PDB at the indicated time points. Dry weight was obtained as the mean value of three parallel cultures. Fungal strains were incubated at 28 °C.

As yeast Snf1 did not show significant effect on vegetative growth, we first tested whether *P. microspora* NK17 *snf1* has a similar role. We cultivated the fungal strains NK17, Δ*snf1* and the complement *snf1*-C on PDA and in PDB. We found that the growth and conidiation of Δ*snf1* decreased remarkably (Figure 2a). The subsequent detection of conidiation and growth curve (based on dry weight of mycelia) also confirmed this phenomenon (Figure 2b,c).

Disruption of *snf1* led to dramatically decreased production of conidia on PDA (Figure 2b). The mutant strain Δ*snf1* produced an average of 0.87 ± 0.15 × 10^6^ (*p* < 0.01) conidia per plate, whereas NK17 produced about 5.74 ± 0.35 × 10^6^ (*p* < 0.01) conidia per plate and the complement strain *snf1*-C restored the number of conidia to 5.93 ± 0.32 × 10^6^ (*p* < 0.01) conidia per plate. Furthermore, compared to NK17, Δ*snf1* produced much less mycelium and exhibited lighter colonies (Figure 2a,c). Thus, *snf1* plays a critical role in conidiation, mycelial pigmentation and vegetative growth in *P. microspora* NK17.

### 3.3. Requirement of snf1 for Utilization of Carbon Sources

**Figure 3:**
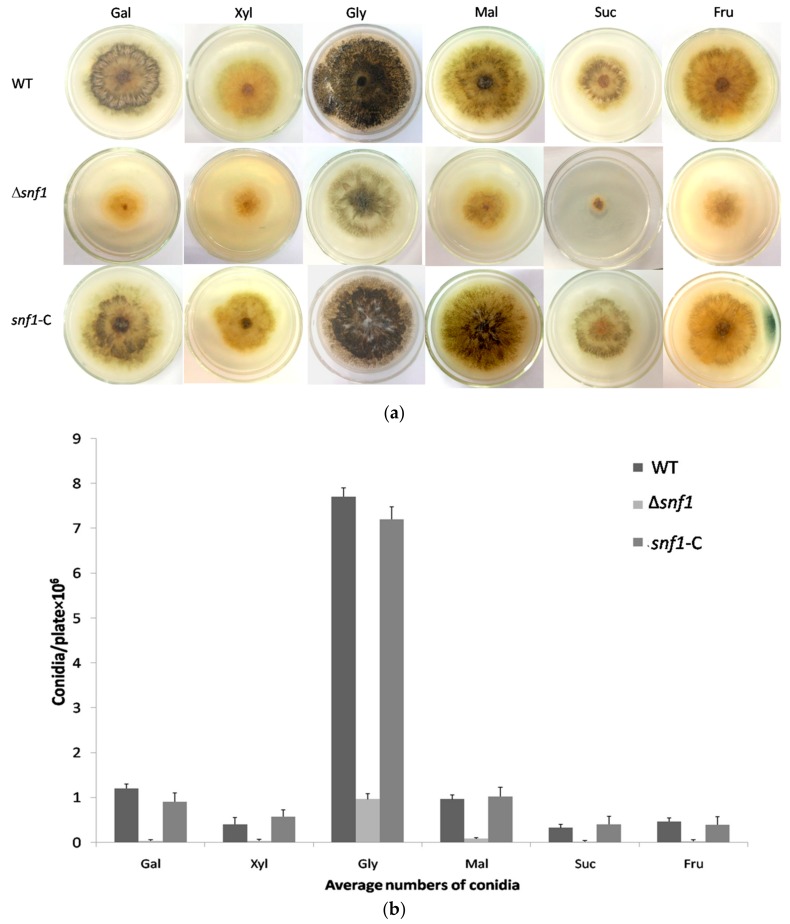
Effect of *snf1* on the use of alternative carbon sources. (**a**) Changes of conidiation/pigmentation and vegetative growth were observed in Δ*snf1* comparing to the wild-type (WT) and the complement strains. The conidiation (indicated by pigmentation) and vegetative growth of Δ*snf1* decreased obviously on sucrose, xylose, fructose, maltose and galactose, while the change was less dramatic on glycerol; (**b**) Numbers of conidia produced by NK17, Δ*snf1* and the complement strain *snf1*-C was determined in the carbon sources as indicated. Triplicate PDA plates for each strain were incubated at 28 °C for 168 h. Error bars represent standard deviations.

Previous studies have established that yeast *snf1* gene is a key regulator in the utilization of secondary carbon sources under glucose-limiting condition. To examine whether *P. microspora* NK17 *snf1* played this canonical role, we tested the growth of Δ*snf1* on galactose, sucrose, glycerol, xylose, fructose and maltose as sole carbon source (Figure 3a). In contrast to the wild type, Δ*snf1* exhibited apparently reduced growth rate on all above carbon sources, in particular, on xylose, fructose, sucrose and galactose. This phenomenon confirmed the classic role of *P. microspora* Snf1 has in the baker’s yeast in sucrose utilization (it was originally named for Sucrose-Non-Fermenting). On the other hand, disruption of *snf1* led to dramatically decrease in conidia production on all the carbon sources (Figure 3b). As we have shown, unlike in the baker’s yeast, *P. microspora snf1* was also needed for the utilization of glucose (Figure 2a), thus, we conclude that Snf1 plays a critical role in the regulation of general carbon metabolism in *P. microspora*.

### 3.4. Roles of Snf1 in Tolerance to External Stress

**Figure 4:**
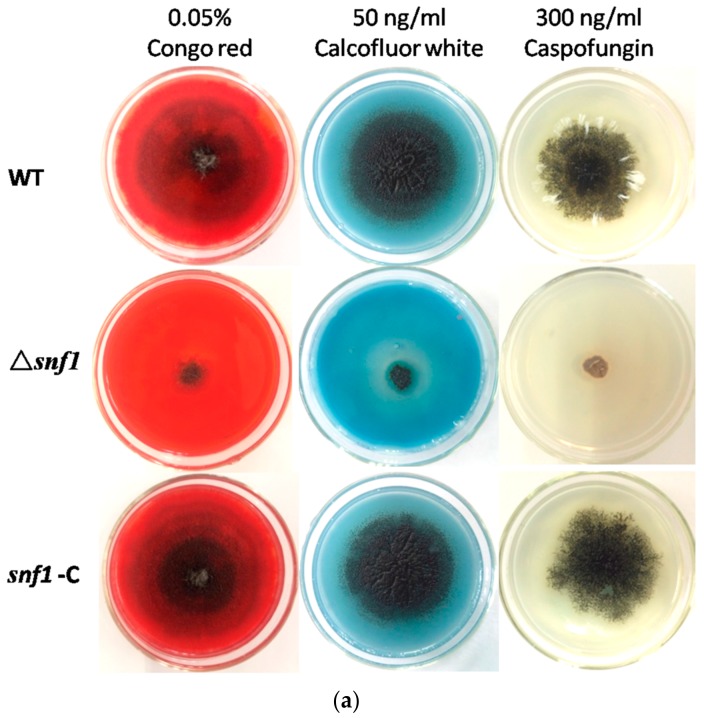

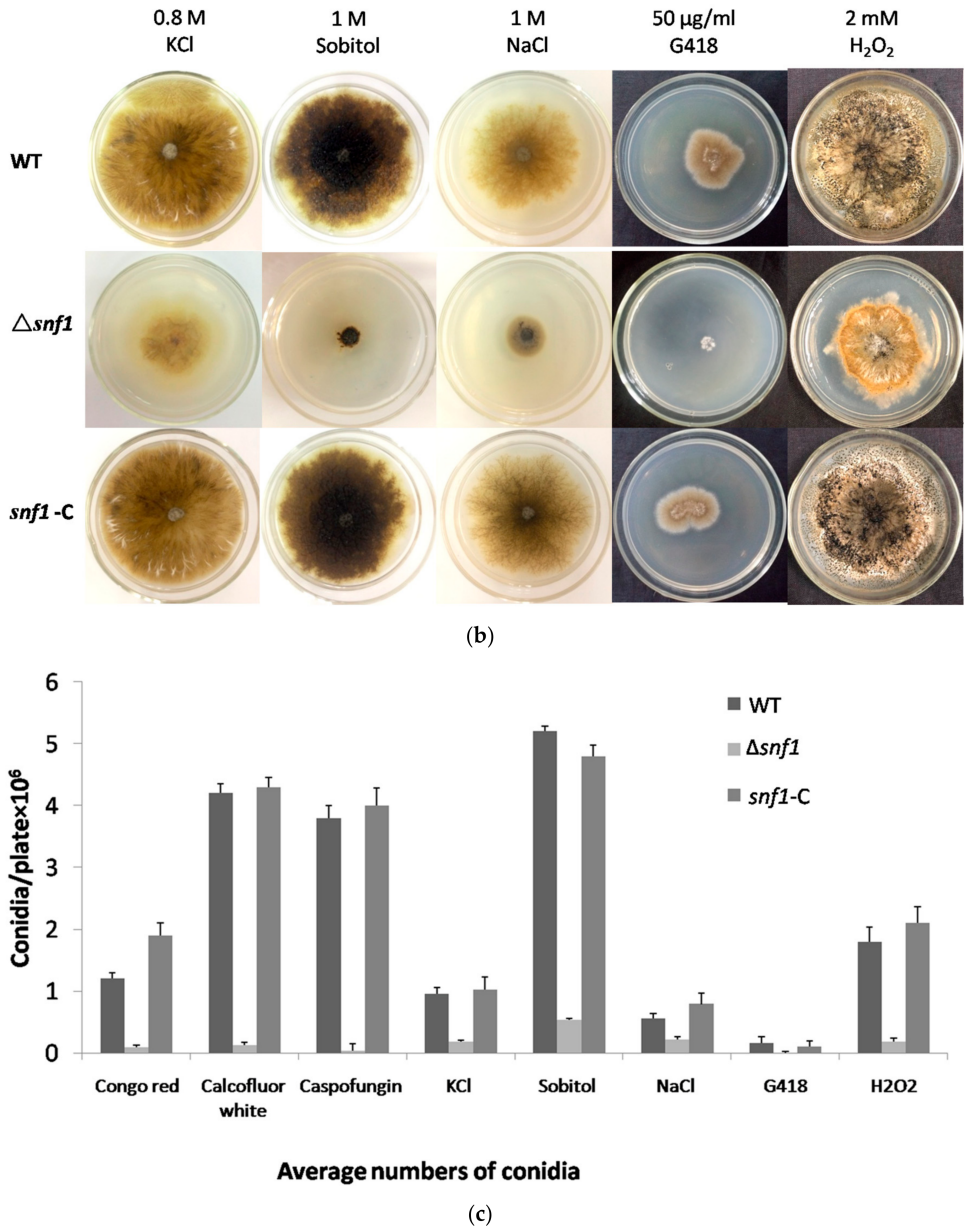
Susceptibility tests for Δ*snf1* to adverse culture conditions. (**a**) Stress tolerance to cell wall inhibitors, Congo red, calcofluor white and caspofungin on PDA. PDA without the supplements served as the control. Conidiation and vegetative growth of Δ*snf1* was significantly affected; (**b**) Stress tolerance to osmotic pressure generated by high concentration of KCl, NaCl and sorbitol. Growth test under oxidative stress (2 mM H_2_O_2_), plus G418 Susceptibility tests for Δ*snf1*; (**c**) The number of conidia produced by NK17, Δ*snf1* and the complementation strain *snf1*-C was determined in the indicated conditions and triplicate PDAs for each strain were incubated at 28 °C for 168 h. Error bars represent standard deviations.

*S. cerevisiae Snf1* plays an important role in stress resistance. To test the role of NK17 *snf1* in response to stress conditions, assays were performed in the presence of the following chemical reagents at appropriate concentrations: cell wall inhibitors Congo red, calcofluor white and caspofungin; osmotic reagents KCl and NaCl; oxidant H_2_O_2_; osmotic stabilizer sorbitol; and the antifungal drug G418. In order to verify whether the deletion of *snf1* affected the integrity of the cell wall, we respectively tested the sensitivity of the mutant strain to three cell wall inhibitors in the medium. As shown in Figure 4a, addition of 0.02% Congo red to PDA plates resulted in profound growth inhibition of Δ*snf1* compared to the controls. Likewise, 50 ng/mL calcofluor white and 300 ng/mL caspofungin also remarkably inhibited the growth of the mutant, while the wild-type and the complement strains underwent a normal growth (Figure 4a, bottom panels). This data clearly demonstrated that cell wall integrity was disrupted in the mutant strain Δ*snf1*. Treatment with high concentrations of salts 0.8 M KCl, 1 M NaCl and 1 M sorbitol, could dramatically inhibit vegetative growth of the mutant strain (Figure 4b), comparing to the control strains (bottom plates in Figure 4b). This result suggests that the cellular responses in NK17 to osmotic pressure requires the function of *snf1.* On plates supplemented with 2 mM oxidant H_2_O_2_, Δ*snf1* obviously showed a slower growth, implying a role of *snf1* in tolerance to this stress condition (Figure 4b). Lastly, the mutant strain Δ*snf1* exhibited a hypersensitivity to 50 μg/mL G418 (Figure 4b). Moreover, disruption of *snf1* also had a significant impact on conidiation in external stress (Figure 4c). 

### 3.5. Sensitivity of Δsnf1 to Heat Shock

**Figure 5:**
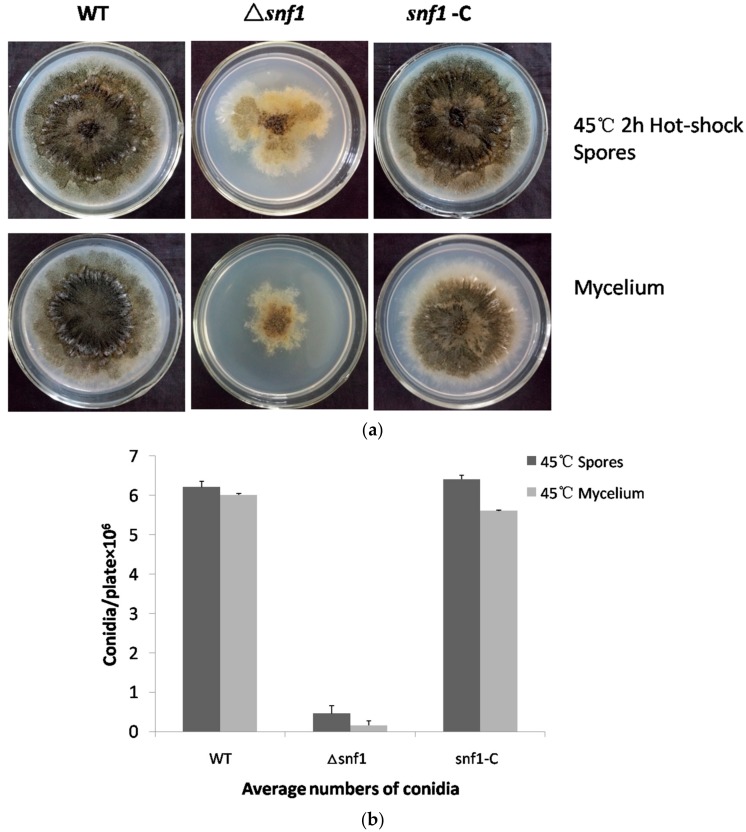
Sensitivity of Δ*snf1* to heat shock. (**a**) Fresh spores and mycelium were incubated in sterile ddH2O at 45 °C for 2 h. Equal numbers of conidia (~5 × 10^4^) or equal volume of mycelium from NK17, Δ*snf1* and the complementation strain *snf1*-C were inoculated on PDA at 28 °C for seven days. Δ*snf1* showed a high sensitivity to heat shock. Conidia without heat-shocked served as control; (**b**) The number of conidia produced by NK17, Δ*snf1* and the complementation strain *snf1*-C was determined in every medium and triplicate PDA plates for each strain were incubated at 28 °C for 168 h and used for quantification. Error bars represent standard deviations.

We tested the growth of Δ*snf1* under heat shock to see whether the gene played any role in this process. The result was shown in Figure 5. As shown, the mutant showed a profound growth defect comparing to the wild-type and the complemented strain (Figure 5a). Moreover, the mutant showed a dramatically decrease in conidiation (Figure 5b). It was indicated that *P. microspora* NK17 Snf1 played a crucial role in heat shock. Interestingly, the mutant spores were more sensitive than the mutant mycelium under incubation at 45 °C for 2 h.

### 3.6. Roles of Snf1 in the Biosynthesis of Secondary Metabolites

**Figure 6:**
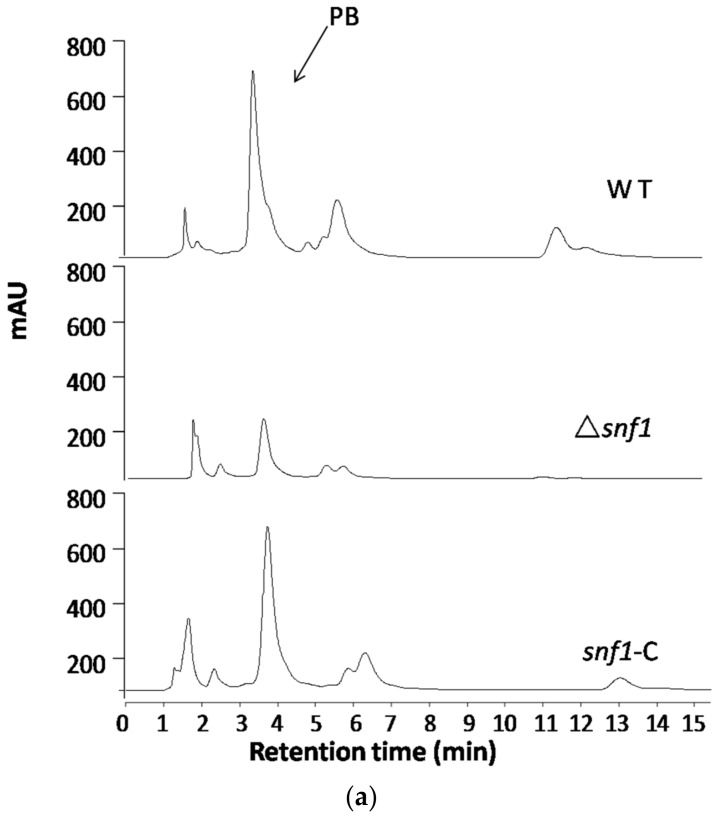

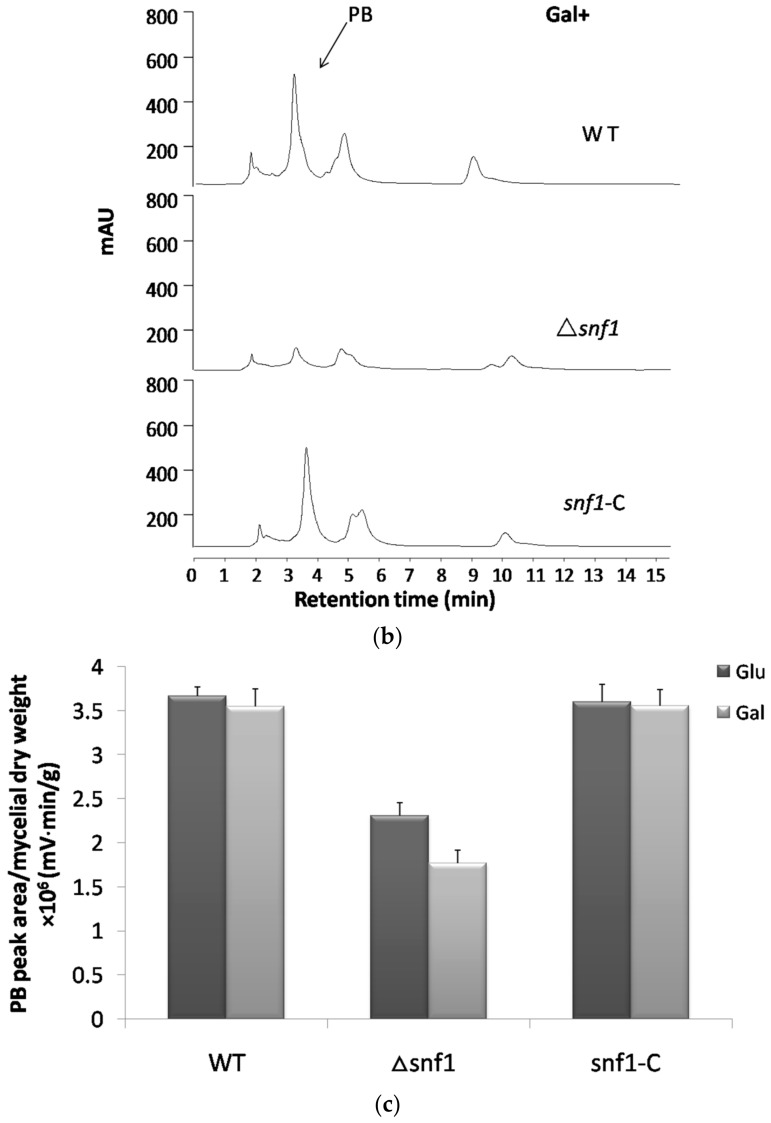
High performance liquid chromatography (HPLC) profiling for secondary metabolites in Δ*snf1*. (**a**) HPLC profiling of the secondary metabolites in Δ*snf1* cultured in PDB. The peak patterns including pestalotiollide B peak of Δ*snf1* was distinct from that of the wild type; (**b**) HPLC profiling of the secondary metabolites in Δ*snf1* in galactose as sole carbon source. HPLC profiling analysis was repeated for three times; (**c**) The ratio of peak area of PB peak relative to the dry weight of mycelium was analyzed. The relative yield of PB in Δ*snf1* significantly decreased comparing with wild-type, especially in the medium of galactose. Error bars represent standard deviations.

Regulation of the biosynthesis of secondary metabolites is complex in filamentous fungi and is usually intimately coordinated with fungal development [24]. To examine the role of Snf1 in biosynthesis of secondary metabolites in *P. microspora* NK17, we examined the profile of secondary compounds in Δ*snf1* cultures by HPLC. We found that deletion of *snf1* obviously altered the profile of secondary metabolites (Figure 6a). Based on HPLC profiling, peaks of most secondary metabolites in Δ*snf1* were substantially lower than that of the wild type. Interestingly, the polyketide pestalotiollide B [24] that we described before was considerably reduced in the mutant strain Δ*snf1* (Figure 6a). Most significantly, when strains were cultured in galactose as sole carbon source, the general production of secondary metabolites in Δ*snf1*, including pestalotiollide B, was almost inhibited (Figure 6b). The production of secondary metabolites was restored to nearly the wild-type level and pattern in the profile of the complement strain (Figure 6b, bottom panel). Above data showed that Δ*snf1* lead to a decrease in mycelium production (Figure 2c), whether it was the determinant of PB change. The ratio of peak area of PB peak relative to the dry weight of mycelium was analyzed (Figure 6c). The result suggested that *snf1* had effects on the production of PB. The above results demonstrate that *snf1* is a regulator of secondary metabolism and may be involved in either activation or silencing of certain gene clusters in NK17.

## 4. Discussion

The Snf1/AMPK is highly conserved among eukaryotic organisms and is an important regulator in carbon metabolism and in energy homeostasis. Many studies have established that Snf1 concerts carbon metabolism by sensing glucose limitation. In *S. cerevisiae*, *SNF1* acts as an activator for more than 400 glucose-repressing genes and some of them are involved in alternative carbon source utilization [25]. Also, other regulatory functions of Snf1 involve in the response to various environmental stress. In this study, we firstly demonstrated Snf1 of *P. microspora* played a comparable role in the utilization of alternative carbon sources. For instance, it is required for the use of sucrose (Figure 2 and Figure 3). However, distinct from the baker’s yeast *snf1*, our data suggest that *P. microspora snf1* took part in the utilization of glucose, as disruption of *snf1* showed a significant delay in growth on PDA plate under permissive condition (Figure 2). This result suggests a complexity of carbon metabolism in fungus [26].

Nonetheless, our observation suggests that *snf1* in *P. microspora* shares similar roles in several other aspects, e.g., in stress response. We found that *P. microspora snf1* was involved in heat tolerance (Figure 5). This function of *snf1* homologs was previously reported in another yeast fungus *Cryptococcus neoformans* [27]. In our fungus, either the spores or the mycelium of the mutant Δ*snf1* was shown to be sensitive to 45 °C heat-shock (Figure 5a). Another important finding is that *snf1* is essential for the cell wall biosynthesis in this filamentous fungus. By challenging the mutant strain Δ*snf1* with the wall inhibitors, the dyes Calcofluor white and Congo, we demonstrated that the growth of the mutant strain was substantially inhibited (Figure 4a). A similar result was obtained for the drug caspofungin which is a chemotherapeutic chemical targeting fungal cell wall biosynthesis. The dyes Calcofluor white and Congo red, which target the biosynthesis of (1, 3) β-glucans and chitin during cell growth and protoplast wall generation, have been used in test of fungal wall integrity [28,29]. Taking into account of this result, we speculated that sensitivity of the mutant strain Δ*snf1* to heat shock might be resulted from the impairment of the cell wall integrity. In addition, previous studies in *S. cerevisiae* have shown that the heat shock transcription factor (HSF) is a target of Snf1 in response to glucose limitation but not to heat shock [30]. Whether HSF is the target of Snf1 is not clear in *P. microspora*. 

Our data suggest that *snf1* is required for conidiation in the filamentous fungus *P. microspora* (Figure 2b and Figure 3b). Disruption of *snf1* in NK17 led to dramatic decrease in conidia production. Although there has been considerable progress in the genetic regulation and light effect on conidiation [31,32], the molecular mechanisms regulating asexual sporulation are still not fully understood in filamentous fungi. Evidence from other study support this view. For example, disruption of *PdSNF1*, a *snf1* homology, also resulted in impaired conidiation and caused malformation of the conidiophore structures in *Penicillium digitatum* [33] and the germination and nucleation of both ascospores and conidia were delayed in *GzSNF1* mutants [34]. Taken together, these data suggest that *snf1* may have a role in concerting the carbon metabolism and conidia development in filamentous fungi, though the underlying mechanism remains to be identified. 

Many filamentous fungi have the capacity to produce a myriad of secondary metabolites. It is still puzzling in many fungi how the synthesis of secondary metabolites is modulated with fungal differentiation and growth, especially with the formation of conidia [24,33]. Fungal secondary metabolism is reportedly regulated by mitogen-activated protein kinase (MAPK) pathway [35], G-protein/cAMP/PKA pathway [36] and velvet complex pathway [32,37]. In this study, we found a possible regulatory role of *snf1* in the production of secondary metabolites. Through HPLC profiling for Δ*snf1*, it was revealed that *snf1* participates in the regulation of the biosynthesis of a polyketide metabolite pestalotiollide B (Figure 6a). Besides, the general pattern of the peaks for products declined sharply in the HPLC profile in the mutant strain Δ*snf1* compared to the wild type and the complement strain. Therefore, this result suggests that Snf1 likely plays a positive regulation in the biosynthesis of these metabolites including pestalotiollide B (PB) (Figure 6c). Notably, in the complement strain, production of PB seems to be restored to the wild-type level. However, this strain displayed a different secondary production profile from the wild type (Figure 6c). This is perhaps caused by the ectopic insertion of the reintroduced copy of *snf1*, which affect the way of its expression. Still more, when the strains were grown in galactose as the sole carbon source, deletion of *snf1* almost abolished the biosynthesis of secondary metabolites (Figure 6b). The above results clearly suggest that *snf1* can function as a critical global regulator controlling secondary metabolism. 

## 5. Conclusions

Our work identified and characterized a SNF1/AMPK homolog in the taxol-producing fungus *Pestalotiopsis microspora*. Snf1 plays a critical role in the regulation of the stress response, alternative carbon source utilization and thermotolerance. Importantly, Snf1 is a key regulator in the growth, development and the biosynthesis of secondary metabolites of filamentous fungi.

## Figures and Tables

**Table 1:** Primers used in this study.

Primer	Sequence (5′—3′)
Snf1-up(F)	GGGGACAGCTTTCTTGTACAAAGTGGAA TCATCAGGCTCGTTATTG
Snf1-up(R)	GGGGACTGCTTTTTTGTACAAACTTGT GTAGGTTCTCGGGTTTCA
Snf1-down(F)	GGGGACAACTTTGTATAGAAAAGTTGTT AGGCGATTCTATTTCTGT
Snf1-down(R)	GGGGACAACTTTGTATAATAAAGTTGT TAACTGCTCGTGTTCTGG
Snf1(F)	TTACCGTCCATCCACAAGAT
Snf1(R)	AAGGGTCTGGGTGGTATGAT
Snf1-PRO(F)	GGGGACAGCTTTCTTGTACAAAGTGGAAATCATACCACCCAGACCCTT
Snf1-TER(R)	GGGGACAACTTTGTATAATAAAGTTGT ATCTTGTGGATGGACGGTAA
Ura3(F)	CGAGGTCGACATAACTTCGT
Ura3(R)	ACGAAGTTATTTCACTGGCA
Hyg(F)	GCCCTTCCTCCCTTTATT
Hyg(R)	TGTTGGCGACCTCGTATT

F: Forward; R: reverse.
